# Ecological interactions among *Saccharomyces cerevisiae* strains: insight into the dominance phenomenon

**DOI:** 10.1038/srep43603

**Published:** 2017-03-07

**Authors:** Roberto Pérez-Torrado, Kalliopi Rantsiou, Benedeta Perrone, Elisabeth Navarro-Tapia, Amparo Querol, Luca Cocolin

**Affiliations:** 1 Instituto de Agroquímica y Tecnología de los Alimentos, IATA-CSIC. Food Biotechnology Department. Institute of Agrochemistry and Food Technology (IATA-CSIC) Avda. Agustín Escardino, 7. E-46980 Paterna (Valencia), Spain; 2 Dipartimento di Scienze Agrarie, Forestali e Alimentari, Università degli Studi di Torino, Largo Paolo Braccini 2, 10095 Grugliasco, Italy

## Abstract

This study investigates the behaviour of *Saccharomyces cerevisiae* strains, in order to obtain insight into the intraspecies competition taking place in mixed populations of this species. Two strains of *S. cerevisiae,* one dominant and one non-dominant, were labelled and mixed, and individual fermentations were set up to study the transcriptomes of the strains by means of RNA-seq. The results obtained suggest that cell-to-cell contact and aggregation, which are driven by the expression of genes that are associated with the cell surface, are indispensable conditions for the achievement of dominance. Observations on mixed aggregates, made up of cells of both strains, which were detected by means of flow cytometry, have confirmed the transcriptomic data. Furthermore, overexpression of the *SSU1* gene, which encodes for a transporter that confers resistance to sulphites, provides an ecological advantage to the dominant strain. A mechanistic model is proposed that sheds light on the dominance phenomenon between different strains of the *S. cerevisiae* species. The collected data suggest that cell-to-cell contact, together with differential sulphite production and resistance is important in determining the dominance of one strain over another.

Intra-species dominance can be defined as the phenomenon that is observed in mixed microbial populations when one individual (strain) is outnumbered by another (belonging to the same species). This is a phenomenon often observed in alcoholic fermentations for the production of wine; at the beginning of the fermentation, mixed populations, composed of more than one strain of *S. cerevisiae,* are present in the must. By the end of the fermentation, a limited number of strains, not uncommonly a single one, are detected. This observation cannot be solely attributed to competition (for nutrients, space) or differences in fitness (resistance to ethanol or other metabolites, differences in growth rate) since such mechanisms do not fully explain the dominance observed. Dominance can also be observed in populations composed of different species. Yeast inter-species dominance is well documented in fermenting musts and possible mechanisms responsible for it have been proposed^1^. In alcoholic fermentations, *S. cerevisiae* yeast can dominate over other non-*Saccharomyces* species^2^. *S. cerevisiae* is able to generate compounds that are toxic to other cells, such as ethanol and SO_2_ as well as antimicrobial molecules, such as GAPDH-derived peptides^3^,^4^,^5^,^6^. In the wine fermentation environment, the production of SO_2_ and the resistance to it, are of particular relevance, and the addition of exogenous SO_2_ is common practice in winemaking to help eliminate undesired autochthonous yeasts. In addition, physical contact between different species has been shown to play an important role in dominance^7^,^8^,^9^. For example, the death of *Lachanchea thermotolerans,* which competes with *S. cerevisiae,* has been found to depend on cell-to-cell contact, and on an unknown antimicrobial peptide^9^. Finally, other physical interactions, involved in dominance of *S. cerevisiae*, have been described in co-cultures with *Kluyveromyces thermotolerans* or *Torulaspora delbrueckii*^7^. In contrast, knowledge regarding intra-species dominance is rather limited and no hypothesis has been put forward that could explain it.

In a previous study, we have investigated the intra- *S. cerevisiae* dominance phenomenon on a collection of well-characterized strains possessing interesting enological features^10^. In that study we identified one strain that was able to dominate, and a second one that in co-culture with other strains repeatedly failed to dominate. Moreover, it was shown that physical contact between the strains was a prerequisite to observe dominance^10^.

The goal of this paper is to further investigate the dominance within the species of *S. cerevisiae* in order to propose a mechanistic model that explains the phenomenon. Our hypothesis is that differences in gene expression between a dominant and a non-dominant strain would identify key genes involved in the process of dominance and could indicate the molecular mechanism underlying such process. Therefore we sought to study and compare the whole transcriptome of a dominant and a non-dominant *S. cerevisiae* strain in single and mixed culture. Furthermore, flow cytometry was employed to elucidate the role of cell to cell contact on dominance.

## Results

### Dominant and non-dominant population growth in natural must

**Figure 1:**
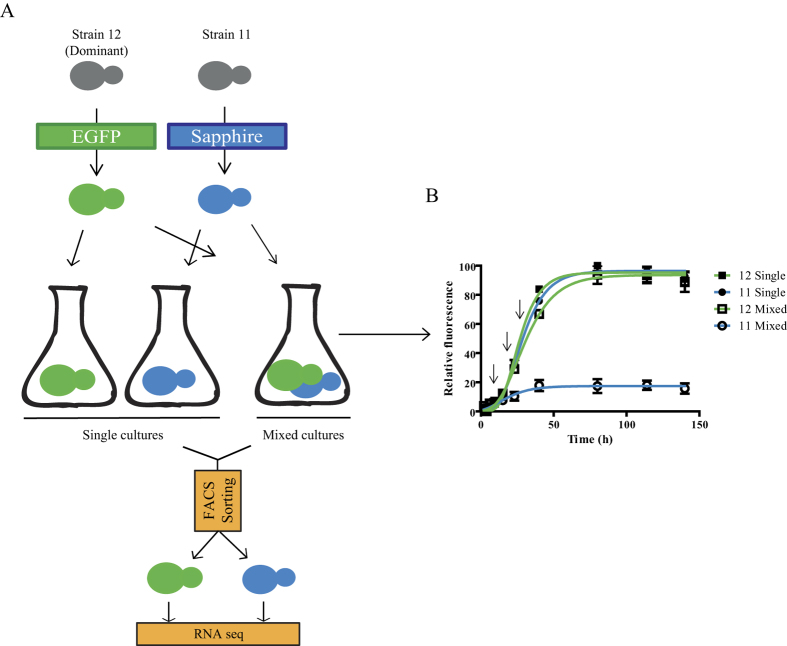
Transcriptomics of the complex dominance phenomenon in yeast. (**A**) A schematic representation of the steps that were followed to determine changes in the transcriptome of cells in single or mixed cultures. (**B**) Evaluation of the yeast growth of fluorescently labelled dominant (12) and non-dominant (11) strains in single or mixed cultures in microtiter plates. The average value of the biological triplicates and standard deviation are shown.

The growth and dominance of *S. cerevisiae*, which had previously been observed in mixed cultures^10^, were studied in microtiter plates. For this purpose, the dominant and non-dominant strains were fluorescently labelled. Possible effects of the labelling on the fitness of the two strains were excluded since no significant differences (*p* < 0.05) were observed in the specific growth rate or lag phase between labelled and wild type strains (results not shown). The growth of each strain was then observed in pure and mixed cultures. As can be seen in Fig. 1B, strain 11 (non-dominant) was affected to a great extent by the presence of strain 12 (dominant). The final fluorescent intensity reached by this strain in the mixed fermentation was significantly lower (tested at a significance level of *p* < 0.005) than that of the pure strain 11 culture. No significant differences were observed between the final fluorescent intensity that was reached in the single and mixed cultures of strain 12.

### Transcriptome analysis in single and mixed cultures in natural must

In order to explain the observed dominance phenomenon, a study was performed on changes in the two strains, at a transcriptomic level, on mixed and single cultures of fluorescently labelled strains in natural grape must. The cultures were sampled at three different times, which are indicated with arrows in Fig. 1B. The different sampling times were chosen to represent different phases in the strain population dynamics: when both strains were present (12 h), when dominant strain 12 started to outstrip non-dominant strain 11 (20 h) and when the non-dominant strain was difficult to detect by means of molecular methods (30 h)^10^. The strains were separated at each sampling time by means of flow cytometry, and their RNA was extracted and sequenced. The raw sequencing data were analysed, as described in the materials and methods section, and two different comparisons were then performed.

#### Monoculture vs mixed culture comparison

**Figure 2:**
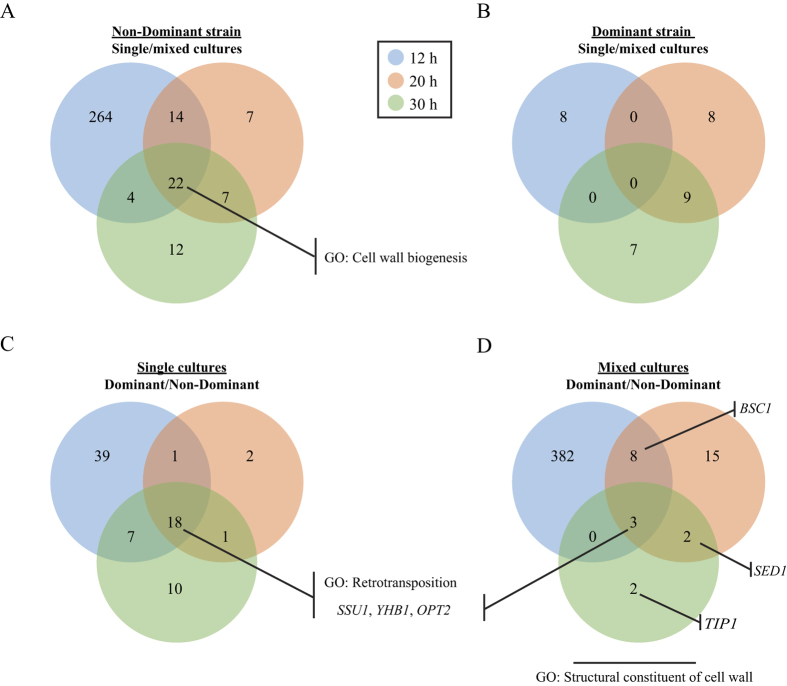
Differentially expressed genes in the dominant and non-dominant strains in single and mixed cultures. Significantly altered gene numbers are represented in Venn diagrams for each considered time (12, 20 and 30 h). Two different types of comparisons were made to analyse the data: Non-dominant strain (**A**) and dominant strain (**B**) in a single culture versus mixed fermentations at each considered time; and dominant strain versus non-dominant strain in single (**C**) or mixed (**D**) cultures at each considered time. The remarkable genes and significant GOs for a specific set of genes are also indicated.

Each single strain culture was compared with the mixed culture at each time (Fig. 2, panels A and B); both up and downregulated genes were considered in each case. This comparison revealed that the non-dominant strain altered a high number of genes (330), due to the competitive condition, whereas the dominant strain was altered much less, and only 32 genes changed expression, some of the changes being related to alcoholic fermentation. Furthermore, the modified expression profile of the non-dominant strain was mainly observed at the first time-point considered (304 genes with modified expression at 12 hours), while a rather limited number of genes showed any modification in the expression for the subsequent time-points (50 genes at 20 hours and 45 genes at 30 hours). The dominant strain presented modified expression of 8 genes at 12 hours, 17 genes at 20 hours and 16 genes at 30 hours. In the non-dominant strain (Fig. 2A), a total of 22 genes were affected at all time-points considered, and these changes were related to cell-wall biogenesis. These genes were upregulated in the mixed culture compared to the single culture. In order to extend the analysis and better understand the specific response of the non-dominant strain, we focused on the genes that showed differential expression in more than one time-points. In this way we obtained a pool of genes that consistently had differential expression and we excluded genes that showed transient modification of their expression. From this pool of genes we further selected those that were not altered in the dominant strain. A specific activation was found for 28 genes in the non-dominant strain in mixed culture (Table 1). Interestingly, the *CRZ1* transcription factor, which is activated by the calmodulin pathway, in response to pheromones and environmental stress, was found to be on. Furthermore, 19 genes (71% of the activated genes) were calmodulin pathway targets. In addition, cell-wall associated proteins, correlated to stress conditions (*YPS3* and *HSP150*), or to yet unknown functions (*PST1* and *YJR061W*), were overexpressed by the non-dominant strain in the mixed culture.

#### Dominant vs non-dominant strain comparison

In this case the dominant strain was compared with the non-dominant strain in the single (Fig. 2C) and mixed (Fig. 2D) cultures at each time (additional information on the deposited data). In this comparison, it was observed that the differential expression between the two strains was much higher in the mixed cultures (412 genes, Fig. 2D) than in the single cultures (78 genes, Fig. 2C). The differential expression in single cultures could be attributed to inherent differences in the genetic make up of the two strains. Eighteen genes were differentially expressed at all the considered times points (Fig. 2C) and 13 of them were retrotransposon genes, which can vary to a great extent among different *S. cerevisiae* strains^11^.

The analysis of the altered genes in the mixed cultures (Fig. 2D) revealed that three genes, namely *SSU1, YHB1* and *OPT2*, were expressed more in the dominant strain than in the non-dominant one, and this was also verified when the strains were compared in the single culture (Fig. 2C). This observation indicates a constant physiological difference between the strains in any condition. Interestingly, when the functional categories of the genes that were significantly altered in the mixed cultures at two of the considered times at least were analysed (Fig. 2D), a statistical significance (*p* value < 2.2 10^−3^) was determined for the GO Structural component of the cell surface (Suppl. Table 1). This was not observed in the pure cultures (Fig. 2C). The activation of a key cell-surface component in the dominant strain, when the cells were incubated in the mixed cultures (Fig. 2D), namely *BSC1*, which encodes for a protein of unconfirmed function, is worth mentioning. This component is similar to cell-surface flocculin Flo11p, *SED1*, which encodes for a structural GPI glycoprotein located in the cell surface, and *TIP1*, which encodes for a major cell wall mannoprotein.

### Role of SO_2_ production and toxicity in the dominance phenomenon

**Figure 3:**
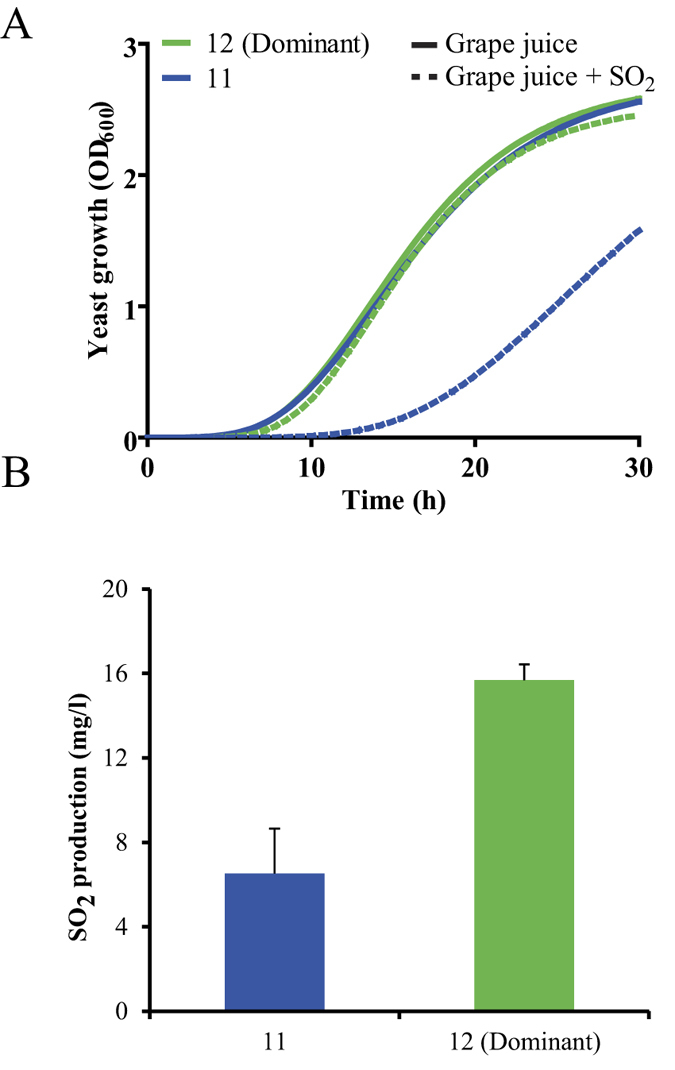
SO_2_ production and resistance in dominant and non-dominant strains. (**A**) Strains, pre-grown in YPD with geneticin, were inoculated into grape juice with (dotted lines) or without 60 mg/l SO_2_ (continuous lines) to evaluate the SO_2_ resistance, and growth was monitored spectrophotometrically according to the time. The average value of the biological triplicates is shown, and the standard deviation was below 10%. Strains containing empty plasmid were used as a control. (**B**) SO_2_ production, after grape juice fermentation, was measured in the dominant and non-dominant strains. The average values of the biological triplicates and the standard deviation are shown. The differences between the two strains were statistically significant (p < 0.05, Fisher exact test).

Since the *SSU1* gene, a crucial gene in resisting SO_2_ toxicity, was overexpressed in the dominant strain we sought to evaluate a possible involvement of strain-dependent SO_2_ production and sensitivity differences in the dominance phenomenon. The growth of the dominant and non-dominant strains was first measured in grape must, and in grape must supplemented with 60 mg/L of SO_2_ (Fig. 3A). The two strains presented very similar growth curves in the grape must without SO_2_. Conversely, when SO_2_ was present, the non-dominant strain growth was affected to a great extent. The ability of the two strains to produce SO_2_ during grape must fermentation was also investigated. As shown in Fig. 3B, both strains were able to produce SO_2_, although the dominant strain produced 2.7 times more than the non-dominant one.

### Cell-aggregation assay

**Figure 4:**
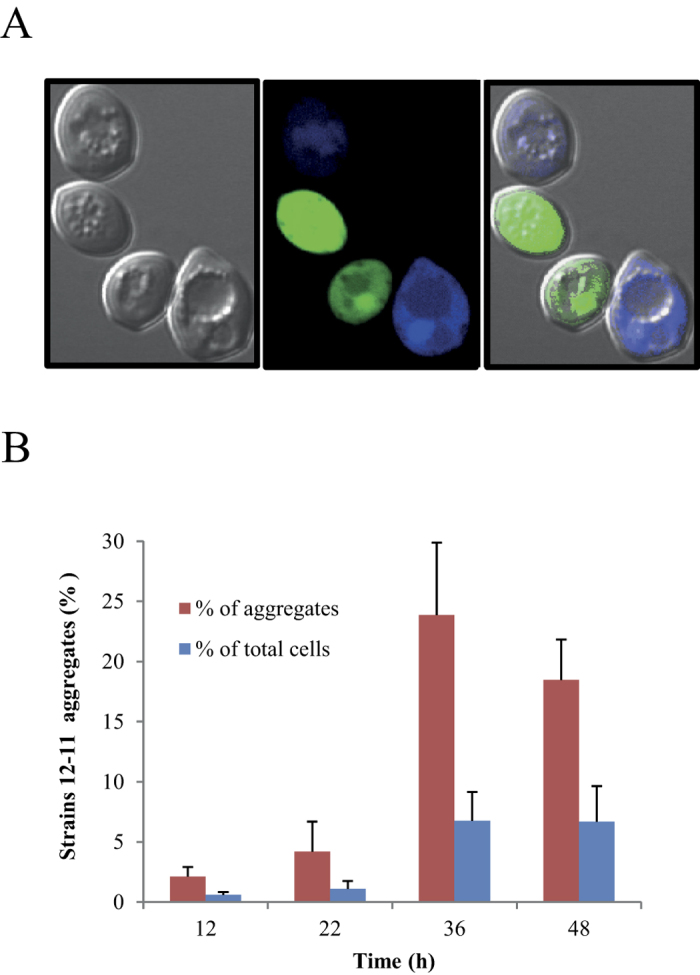
Cell aggregation in natural must. (**A**) The presence of aggregates containing cells of both the dominant and non-dominant strains was observed by means of fluorescence microscopy and using the previously described fluorescence labelled strains. (**B**) The aggregates that contained cells from both strains were quantified at different times by means of flux cytometry and compared to the total number of cells and to the total number of aggregates. The average values of the biological triplicates and the standard deviation are shown. The 36 h and 48 h times showed a significant difference for both parameters (p < 0.05, Fisher exact test), compared to the first considered time (12 h).

As reported above, genes related to the cell surface were differentially overexpressed when the cells were in the mixed cultures, and the cell-to-cell contact had previously been suggested as a driving force that could explain the dominance between different species^7^,^8^,^9^. We therefore studied the cell-to-cell contact between the dominant and non-dominant strain, looking into the formation of cellular aggregates. Fluorescence microscopy was used first to visualize any inter-strain contacts under the conditions that could determine the previously observed dominance. An example of the cell-to-cell contact between the dominant and the non-dominant strain is shown in Fig. 4A. Flux cytometry was then used to quantify cell aggregates that had formed under the same conditions (Fig. 4B). The results showed inter-strain aggregates were formed and a statistically significant increase in the percent of the inter-strain aggregates was observed between the 12 h time point and the 36 and 48 time points. It has to be noted that there is a time delay between the massive gene expression changes observed for the non-dominant strain (peaked at 12 h) and the phenotypic response (cell-aggregation) that peaked between 36 and 48 h. Nevertheless, as can be observed in Fig. 1B, a clear inhibition of the non-dominant strain was established at 30 h. Furthermore, three genes encoding for cell wall/surface associated proteins, namely *BSC1, SED1* and *TIP1*, showed increased expression in the dominant strain, mainly at 20 and/or 36 h in the mixed culture (Fig. 2).

## Discussion

Natural and anthropogenic habitats are influenced to a great extent by the microbial communities that reside within them, and by human activities. The composition of such microbial communities is the result of interactions among the various microorganisms and between these microorganisms and the environment. One very common form of microbial interaction is competition, and this competition can be displayed between members of a community that belong to the same, to similar or to distant taxa. Microbial competition can be manifested in various forms: competition for resources, the production of toxic compounds, or differences in growth behavior and/or fitness.

Alcoholic fermentation for the production of wine is a biological process in which yeast species have specialized. The yeast species *S. cerevisiae* is exceptionally well adapted in the environment that implies the transformation of grape into (fermenting) must. Although the grape surface is populated by an assortment of yeast species, which inevitably pass to the grape must, *S. cerevisiae* quickly dominates fermenting must, and other species are often undetectable after the first days of fermentation^12^. This dominance has been attributed to the fitness advantage of *S. cerevisiae,* due to the high transformation efficiency of sugar into ethanol, with concomitant heat production, that inhibit non-*Saccharomyces* species as well as high resistance to the hostile grape must environment (pH, organic acids, SO_2_ and lack of oxygen)^13^,^14^. Moreover, non-*S. cerevisiae* species are more susceptible to ethanol and other environmental parameters, and are therefore not competitive. Recently, another form of microbial interaction, mediated by cell-to-cell contact, has been shown to be important in shaping the composition of microbial communities in fermenting must, and in the dominance of *S. cerevisiae* over other wine yeast species^2^,^15^.

Although interspecies competition has been studied extensively in various habitats, including in the food (e.g. mediated by bacteriocins, and during alcoholic fermentation), medical and agricultural fields^16^, intraspecies competition, i. e. competition between individuals of the same species, has received limited attention. This aspect has recently been addressed in the wine fermentation field and it has been shown that dominance of one *S. cerevisiae* strain over another cannot be explained solely by the presence of a diffusible molecule, and that this process probably involves cell-to-cell contact^10^.

An important observation has been made, related to the massive number of genes with modified and mostly upregulated expression levels in the non-dominant strain, in the presence of the dominant strain. This modified gene expression was also reflected in the comparison of the expression profile between the dominant and non-dominant strains. From this comparison, it was possible to deduce that most of the genes differentially expressed between the two strains in the mixed culture were overexpressed in the non-dominant strain. The genes involved in the calmodulin pathway were among the modified genes of notable interest. *CRZ1* has been found to encode for a calcineurin-dependent transcriptional factor that activates target genes in response to environmental stresses, mediated by Ca^2+^-calmodulin^17^. In this study, it was shown that *CRZ1* regulation takes place at the transcription level; the non-dominant strain showed an increased mRNA level when it was co-cultured with the dominant strain. Interestingly, it had previously been shown that the Celcineurin/Crz1p signaling pathway plays a role as a regulator of the response to cell wall damage^18^. A series of genes, namely *CMK2, HSP150, PHO84, PRM10, PST1, YJL171C, YPS3,* which were upregulated in conditions of transient cell damage^18^, were also upregulated in the non-dominant strain (Table 1). Taken all together, these observations suggest that the non-dominant strain, in a co-culture with a dominant strain is subjected to cell wall stress/damage and at least partially activated pathways, related to cell-wall organization and biogenesis.

A rather different response was exhibited by the dominant strain in the presence of the non-dominant strain. As can be seen in Fig. 2B, only a limited number of genes showed a modified expression for the dominant strain. By comparing the expression profile of the dominant and non-dominant strains (Figs 3C,D), the different genetic make-up of the two strains became evident. The important ecological advantage of the dominant strain in the fermenting must environment can be associated with the increased expression of the *SSU1* gene, which has been shown to be responsible for an increased resistance to SO_2_^19^. The addition of SO_2,_ during or after grape crushing, is an enological practice that is commonly adopted to provide protection against oxidation and the growth of undesired microbial species^20^, because it acts as a strong selective agent. *S. cerevisiae* is known to be among the most resistant yeast species found in grapes and musts^21^. The *SSU1* in *S. cerevisiae,* which encodes a sulphite efflux pump, confers increased resistance to SO_2_^19^. The endogenous production of sulphite by *S. cerevisiae* has also been described^22^. The results of the physiological tests in this study (resistance to SO_2_ and production of SO_2_), taken together with the increased *SSU1* expression in the dominant strain, would seem to support the hypothesis that the dominant strain is highly competitive in grape must due to the increased SO_2_ resistance.

**Figure 5:**
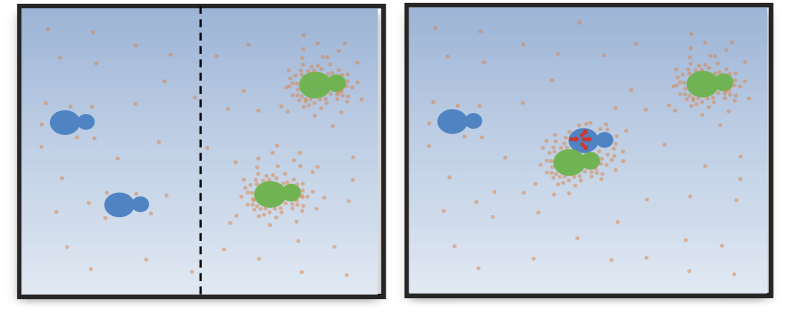
Schematic representation of the proposed model set up for the complex dominance phenomenon among the *S. cerevisiae* strains. The panel on the left represents the growth of the dominant (blue cells) and non-dominant (green cells) strains, when separated by a 0.45 μm membrane, where no dominance was observed, even though SO_2_ (cream dots) was able to diffuse. The panel on the right shows that the cells were able to form aggregates, and that the microenvironment in the proximity of the dominant strain, which produced greater amounts of SO_2_, generated a stress response (red arrows), which was mediated by calmodulin, and a reduced growth, which ended with one strain being dominant over the other.

However, the dominance phenomenon that has been investigated here cannot be explained completely by differences in SO_2_ resistance. When the two strains share the same grape must, but are physically separated by a membrane, both grow equally, and no dominance is observed^10^. This is clear evidence that suggests an important role of cell-to-cell contact in intra-species competition. Cell-to-cell contact and the subsequent cell aggregation have been studied extensively in yeasts, and are associated with mating, biofilm formation, flocculation and the formation of hyphae^23^. Cell aggregation (co-flocculation) between different yeast species has recently been proposed as a mechanism that could govern population dynamics in complex ecosystems, such as wine^15^. The genetic determinants that are responsible for cell adhesion and aggregation in *S. cerevisiae* have been identified and encode for cell-wall associated proteins that have the ability to recognize amino acid or sugar residues on the cell surface of neighbouring cells^24^. In this study, 2 genes encoding GPI-anchored cell wall proteins, as well as a third gene similar to *FLO11*, a known adhesin/flocculin encoding gene^24^, were upregulated in the dominant strain when co-cultured with the non-dominant one. This fact, together with the significant modification of the cell-wall associated genes in the non-dominant strain, led us to hypothesize the role of cell aggregation in the intra-species competition and dominance processes. The inter-strain cell aggregates were tested, by means of flow cytometry, when the two strains were co-cultured. Mixed aggregates, composed of cells belonging to both the dominant and non-dominant strains, were observed. The formation of mixed aggregates peaked at around 36 hours of the co-culture, and represented 25% of the total cell aggregates that were formed. Therefore, a mechanistic model, in which the combination of the simultaneous action of *SSU1,* and several cell surface proteins that are considered to lead to aggregation, can be proposed (Fig. 5). The manifestation of dominance is the result of the effect of the proximity of the dominant and non-dominant strain cells, which increases the SO_2_ stress and creates microevironments in the medium surrounding the cells that are not favourable for the growth of the non-dominant strain.

To the best of the authors’ knowledge, this is the first attempt that has been made to obtain a detailed understanding of the inter-strain competion and dominance phenomenon which is known to take place in nature. An innovative approach has been used to tackle the methodological problems related to the transcriptomic study of two closely related microrganisms. The results obtained suggest an important role of cell aggregation in the competition that takes place between the representatives of one species. To consolidate the proposed model it is important to understand whether the observations of this study are extended to other members of the species *S. cerevisiae*, i.e. strains that form inter-strain aggregates dominate in mixed populations. Elucidation of the role of the genes *SED1, BSC1, TIP1* in the formation of inter-strain aggregates would provide further evidence in support of the proposed model.

## Materials and Methods

### Yeast strains and media

The *S. cerevisiae* wine yeast strains used in this study have been previously described in Perrone *et al*.^10^ as strain 11 (non-dominant) and strain 12 (dominant). The BY4741 laboratory strain (*MATa his3*∆0 *leu2*∆0 *met15*∆0 *ura3*∆, Open Biosystems) was also used. The yeasts were grown and maintained in YPD (2% glucose, 2% peptone, 1% yeast extract, all from Oxoid, Milan, Italy) with G418 (200 μg/mL, Sigma, Milan, Italy), when required. Nebbiolo must was used for the fermentation comparisons, and 60 mg/L SO_2_ was added when necessary.

### Plasmid and strain construction

The BY4741 laboratory strain was transformed with double digested pKT150 and pKT127 plasmids (4894 bp), and a cassette containing a GPD promoter sequence. pKT127/150 plasmids contain the *KANMX* gene, which was used as a transformation marker to select the G418 resistant *S. cerevisiae* transformants. Plasmids, containing Sapphire Fluorescent Protein (eSapphire) and Green Fluorescent Protein (GFP), respectively, were cut with *Sal*I and *Pst*I to facilitate plasmid recombination. Promoter manipulations were carried out by inserting the desired promoter upstream of the GFP/eSapphire coding region. The GPD promoter sequence was amplified from a p416-GPD plasmid. Amplification of the GPD promoter was performed with the following designed primers: PromPktF (TGACACTATAGAACGCGGCCGCCAGCTGAAGCTTCTACGTTTTGCTGGCCGCATCTTCTCA) and PromPktR (ATTGGGACAACACCAGTGAATAATTCTTCACCTTTAGACATATCCGTCGAAACTAAGTTGTGGTTG), which are composed of 40 base pairs that are complementary to pkt150/127 plasmids for integration within 4860 bp and 44 bp, and 20 base pairs for the amplification of the GPD promoter from p416GPD. The GPD sequence (4664 bp) was amplified from 4562 bp to 3448 bp, using Phusion High-Fidelity DNA Polymerase (New England Niolabs, Barcelona). Yeast transformation was performed using the LiAc/SS Carrier DNA/PEG method, as described by Gietz *et al*.^25^. Double digested plasmids and a cassette containing the promoter were added in a proportion of 1:3. The labelled-by4741 strain was selected in the Yeast Nitrogen Base, minus Ura (the *URA3* region belonging to p416-GPD), with G418 100 μg/ml (the *KANMX* sequence belonging to the pKT 150 and pKT 127 plasmids). The recombined plasmid was rescued using a colony plasmid rescue protocol adapted by Hoffman and Winston^26^, and amplified in *E. coli* electrocompetent cells (DH10B strain), prepared previously as described by Sambrook and Russel^27^. After purification, the GPD promoter-GFP/eSapphire cassette was amplified. A GenElute Plasmid miniprep kit (Sigma) was used to recover the recombined plasmid from *E. coli,* transformed by means of electroporation, according to the manufacturer’s instructions, for the DH10B strain (1700 V). The transformants were grown for 24 hours in LB broth with ampicillin (100 μg/ml), before the extraction of the plasmid. The purified plasmid was subjected to PCR with the following primers: ScProFluoF (TAATAACAATGTGCAAATAAAAAACTATCTTACAGGCAATTAATAAGTATATAAAGACGGTAGGT), and ScFluoR (ATTGGGACAACACCAGTGAATAATTCTTACCTTTAGACATATCCGTCGAAACTAAGTT). Phusion High-Fidelity DNA Polymerase was used for the amplification of the cassette. The PCR product was integrated in the yeast genome between base pairs 199230 and 199231 of chromosome I, as manipulation in this area of the genome does not affect the fitness of the strains^28^. The forward primer was designed as follows: a tail of 40 bp, complementary to the region upstream of 199230 bp, and 20 bp, complementary to the GPD promoter sequence reverse primer tail, was designed to be complementary to the right side of the genome, and the 20 base pair was designed to be complementary to the terminator (TEFt). The transformants were selected on YPD with G418 (200 μg/mL, Sigma). Integration between 199230 and 199231 bp was verified by means of the following primers: IntVerFluoF (CCGGCAAAACAGCATTCCAGG) and VerFluoR (ACGACCATCCATGGGGTG). The former was designed in the KANMX region of the cassette, and the latter was designed 200 bp, after the integration. PCR was performed with Fermentas Taq DNA Polymerase, and resulted in a PCR product of 995 bp.

### Strain growth and fitness determination

The growth of the fluorescent labelled dominant strain (strain 12) and the non-dominant strain (strain 11) was determined in single and mixed fermentations by measuring the emissions at 520 nm, after excitation at 485 nm, using a POLARStar Galaxy Microplate Reader (BMG Labtech, Offenburg, Germany). In order to test the fitness of the fluorescent labelled strains, and to compare them with those of the wild strains (strains 11 and 12), the growth kinetics was determined, as described by Salvadò *et al*.^29^. Growth was monitored at 600 nm using a SPECTROstar Omega instrument (BMG Labtech, Offenburg, Germany). Measurements were conducted every 30 minutes for 3 days, after a pre-shaking of 30 s. The microplate wells were filled with 0.01 ml of inoculum and 0.49 ml of natural grape must, in order to ensure the continuous presence of an initial OD of approximately 0.05. All the experiments were carried out in triplicate.

### Mixed fermentations, flow cytometry and RNA extraction

Single and mixed fermentations, including strain-dye swapping (the use of strains with changed fluorescent markers, GFP changed to eSapphire, and *vice versa*), were performed in triplicate. The dominant and non-dominant strains were inoculated to reach the same cell density (0.1 OD) in 10 ml of Nebbiolo must (Fig. 1A). Fermentations were carried out at 25 °C, with continuous shaking. Aliquots of 1 ml were taken at 3 phases of the fermentation. The first sample was taken at the beginning of the exponential growth phase, the second in the middle of the exponential growth phase, and the last at the beginning of the stationary phase. The samples were centrifuged rapidly to eliminate the must fractions, and RNA*later* (Ambion, Monza, Italy) was added and immediately frozen in liquid nitrogen. The samples were kept at −80 °C, until the flow cytometry separation process was performed.

Cells were sorted using a MoFlo (Dako cytomation, Glostrup, Denmark) cytometer, according to the fluorescence intensities of the populations, after separating the cells by sonication (40 seconds at 50% intensity) and filtering (Filcon 30 μm, BD Biosciences). The cytometer was equipped with two lasers: 488 nm (for GFP excitation) and 345 nm (for Sapphire excitation). The FL7 570/20 and FL1 530/20 channels were used for GFP and Sapphire, respectively. Separation was performed at a sorting rate of up to 20000 events per second, for the time necessary to reach between 500000 and 1000000 cells. The cells were collected in tubes containing a PBS buffer, and were then put directly into dry ice in order to flash freeze them as they exited from the cytometer.

The frozen samples were kept at −80 °C, and then thawed in ice for the subsequent RNA extraction. The RNA Mini Kit (Qiagen, Madrid, Spain) was used, according to the manufacturer’s instructions. RNA quality was checked using the Bioanalyser (Agilent Technologies, Madrid, Spain), and those samples with a good quality RNA, that is, those that showed 5 as the minimum of the RNA Integrity Number (RIN), were sequenced. Triplicate samples were sequenced for each considered time.

### RNA sequencing and analysis

An Illumina RNA amplification kit (Thermo-Fisher) was used in order to amplify the small amount of RNA extracted from the cells separated by means of cytometry. Sequencing was performed by means of the 5500 SOLiD system (Life Technologies, Madrid, Spain). Seventy-eight million reads per strain were obtained, and short sequences (75 bp) were assembled and aligned using a reference *S. cerevisiae* strain (Saccer2). The values of FPKM (Fragments Per Kilobase of exon per Million fragments mapped) were transformed for the statistical test as log2 (FPKM1/FPKM2) and uploaded on Cuffdiff (Cufflinks V1.3.0, http://cufflinks.cbcb.umd.edu/), where FPKM1 stands for the dominant strain data and FPKM2 stands for the non-dominant strain data. The FPKM parameter was used for pair end RNA-Seq experiments, where fragments were sequenced from both ends, and two reads were performed for each fragment. The Q value, which represents the significance value of the statistical analysis, performed through the use of a cufflink platform, was calculated, and significant transcriptome differences were identified. A functional analysis was performed to search for any significantly overrepresented gene ontologies (GO) in a set of genes, using the Genemania functional network predictive online tool^30^.

### Flow cytometry aggregation tests

The dominant-GFP labelled and non-dominant-eSapphire labelled strain aggregation was tested as pure and mixed growth in must. Four sampling times were chosen (12, 22, 36 and 48 hours) and aggregation was evaluated using a BD FACSVerse flow cytometer from BD biosciences. FITC and V500 filters were used to detect the Green and Sapphire fluorescent proteins, respectively. A P1 region, corresponding to single cell events, was determined by passing a sonicated sample through the cytometer (40 second at an intensity of 50%). Events showing higher size values, corresponding to aggregate events, were collected in a P2 region. The percentages of green, blue and mixed events were calculated within the P2 region.

### SO_2_ determination

The SO_2_ amount was determined through the use of an Enzytec color SO_2_–total kit (BioPharm, Milan, Italy), according to the manufacturer’s instructions.

## Additional Information

**Accession Codes**: The sequence reported in this paper has been deposited in the Gene Expression Omnibus (GEO) database, www.ncbi.nlm.nih.gov/geo (accession no. GSE81270).

**How to cite this article:** Pérez-Torrado, R. *et al*. Ecological interactions among *Saccharomyces cerevisiae* strains: insight into the dominance phenomenon. *Sci. Rep.* **7**, 43603; doi: 10.1038/srep43603 (2017).

**Publisher's note:** Springer Nature remains neutral with regard to jurisdictional claims in published maps and institutional affiliations.

## Supplementary Material

Supplementary Table

## Figures and Tables

**Table 1:** Significantly over-expressed genes at two of the considered times at least in only the non-dominant strain in the mixed versus single fermentations^#^.

Gene	Function	Time (h)			
		12	20	30	Crz/Cmk target
*ADH6*	NADPH-dependent medium chain of alcohol dehydrogenase	1.8	2.4	2.8	
*ADI1*	Acireductone dioxygenease	2.8	3.1	—	yes
*AIM17*	Putative protein of an unknown function	1.7	2.0	2.6	yes
*ALD6*	Cytosolic aldehyde dehydrogenase	2.3	2.2	2.2	yes
*BAG7*	Rho GTPase activating protein (RhoGAP)	3.5	2.5	—	yes
*CAR1*	Arginase	2.9	2.9	3.4	
*CMK2*	Calmodulin-dependent protein kinase	3.2	2.9	3.0	yes
*CRZ1*	Transcription factor involved in the stress response	2.1	2.3	—	yes
*DIA1*	Protein of an unknown function	4.1	3.3	4.8	yes
*DLD3*	D-lactate dehydrogenase	—	4.2	3.0	
*FET4*	Low-affinity Fe(II) transporter of the plasma membrane	3.4	2.3	3.0	yes
*GYP7*	GTPase-activating protein for the Rab yeast family members	—	2.9	3.0	yes
*HAL5*	Putative protein kinase	3.2	2.1	—	yes
*HSP150*	O-mannosylated heat shock protein attached to the cell wall	1.7	1.4	—	yes
*IPT1*	Inositolphosphotransferase	1.5	2.7	2.3	yes
*PEP12*	Target membrane receptor (t-SNARE)	2.4	2.5	—	yes
*PHO84*	High-affinity inorganic phosphate (Pi) transporter	3.2	2.6	—	
*PLB1*	Phospholipase B (lysophospholipase)	2.1	1.6	—	yes
*PMC1*	Vacuolar Ca2+ ATPase	3.4	1.9	—	yes
*PRM10*	Pheromone-regulated protein	3.8	3.1	3.2	yes
*PST1*	Cell-wall protein that contains a putative GPI-attachment site	1.6	2.0	—	
*RCN2*	rotein of an unknown function;	2.9	2.2	2.2	yes
*ROG3*	Protein that binds the ubiquitin ligase Rsp5p	2.2	2.5	—	
*SNZ1*	Protein involved in vitamin B6 biosynthesis	3.6	3.0	—	
*YJL107C*	Putative protein of an unknown function	2.8	3.2	4.5	yes
*YJL171C*	GPI-anchored cell-wall protein of an unknown function	1.5	2.0	—	yes
*YJR061W*	Putative protein of an unknown function	2.3	1.9	2.3	
*YPS3*	Aspartic protease from the cell wall	2.3	3.9	2.6	yes

^#^The results have been log-fold changed.
