# Supplementary material for: Ecological interactions among Saccharomyces cerevisiae strains: insight into the dominance phenomenon
Source: Sci Rep. 2017 Mar 7;7:43603. doi: 10.1038/srep43603 (PMC5339867; doi:10.1038/srep43603)
Supplement: Supplementary Table [file srep43603-s1.pdf]

Ecological interactions among *Saccharomyces cerevisiae* strains: insight into the complex dominance phenomenon

Roberto Pérez-Torrado[a1], Kalliopi Rantsiou[b1], Benedeta Perrone[b], Elisabeth Navarro-Tapia[a], Amparo Querol[a*] and Luca Cocolin[b*]

Supplementary Table 1. Functional categories of genes significantly altered at least in two time points in mixed (A) and singles cultures (B) dominant strain versus non-dominant strain comparisons.

(A)

Function	FDR	Genes in network	Genes in genome
structural constituent of cell wall	0.0022384725191035206	4	12

(B)

Function	FDR	Genes in network	Genes in genome
RNA-directed DNA polymerase activity	2,27E-52	34	45
DNA polymerase activity	1,71E-45	34	61
DNA-directed DNA polymerase activity	1,41E-42	33	59
retrotransposon nucleocapsid	2,27E-38	34	89
ribonuclease activity	5,40E-36	33	89
transposition, RNA-mediated	1,44E-34	34	105
transposition	3,86E-34	34	108
nuclease activity	1,12E-32	35	135
nucleotidyltransferase activity	1,12E-32	34	118
peptidase activity	2,97E-28	33	135
hydrolase activity, acting on ester bonds	1,37E-20	35	281
